# Impact of Maternal Nutritional Status and Mental Health on Children with Obesity: Relationship Between Anthropometric Parameters, Food Addiction, and Stress

**DOI:** 10.3390/ijerph22091312

**Published:** 2025-08-22

**Authors:** Joicy Karla Grangeiro Pereira, Rúbia Cartaxo Squizato de Moraes, Thallyta Alanna Ferreira Viana das Neves, Davyson Barbosa Duarte, Cristiane Cosmo Silva-Luis, Paulo César Trindade da Costa, Carla Lúcio Alves, Melyssa Kellyane Cavalcanti Galdino, Carla Alexandra da Silva Moita Minervino, Nassib Bezerra Bueno, José Luiz de Brito Alves, Vinicius José Baccin Martins

**Affiliations:** 1 Department of Nutrition, Federal University of Paraiba, Joao Pessoa 58051-900, Brazil; joicykarla21@hotmail.com (J.K.G.P.); rubiacartaxo@gmail.com (R.C.S.d.M.); thallytaviana@hotmail.com (T.A.F.V.d.N.); davyson.duarte@ufpe.br (D.B.D.); criscosmosilva@hotmail.com (C.C.S.-L.); paulocesarnutricionista@gmail.com (P.C.T.d.C.); jose.luiz@academico.ufpb.br (J.L.d.B.A.); 2 Department of Psychology, Federal University of Paraiba, Joao Pessoa 58051-900, Brazil; carla.lucio.alves@gmail.com (C.L.A.); melyssa_cavalcanti@hotmail.com (M.K.C.G.); carlamoitaminervino@gmail.com (C.A.d.S.M.M.); 3 Postgradute Program in Nutrition, Faculty of Nutrition, Federal University of Alagoas, Maceio 57072-900, Brazil; nassib.bueno@fanut.ufal.br; 4 Department of Biomedical Sciences, Federal University of Paraiba, Joao Pessoa 58051-900, Brazil

**Keywords:** stress, dyad, food addiction, obese, children

## Abstract

Childhood obesity is a complex and multifactorial disease influenced by various factors including behavioral, physical, and psychological conditions. This cross-sectional study aimed to evaluate the impact of maternal and child stress and food addiction on the nutritional status of children with obesity. Children aged 6 to 12 years were divided into Control Group (n = 42) and Obesity Group (n = 68) according to BMI-Z score. Mothers were allocated to their children’s respective groups. Anthropometric measures, body composition, stress levels, and food addiction were evaluated in both children and mothers. Children with obesity exhibited a significantly higher prevalence of food addiction compared to controls (22.1% vs. 2.6%, *p* = 0.006), while mothers in the control group showed higher stress levels (68.6% vs. 46.3%, *p* = 0.039). Positive correlations were observed between mother–child pairs for weight (r = 0.433, *p* < 0.01) and waist circumference (r = 0.461, *p* < 0.01). In children, food addiction was a significant predictor of BMI-Z scores (adjusted R square = 0.186); however, maternal BMI and stress were more important predictors (adjusted R square = 0.468). These findings highlight the influence of maternal physical and psychological health on childhood BMI-Z scores. Effective interventions should target both mother and child to improve overall health outcomes.

## 1. Introduction

Obesity is a complex multifactorial disease caused by genetic, psychological, nutritional, and metabolic factors that can contribute to the development of non-communicable diseases or, by itself, lead to tissue and organ impaired [1]. In addition to these etiological factors, the nutritional status of parents has been associated with the nutritional status of their children [2]. A 6-year cohort study found that excess BMI before pregnancy, excessive weight gain during pregnancy, and large-gestational age are important predictors of excess weight after delivery [3]. An assessment of parent–adolescent dyads found an association between parental and adolescent weight status, even after adjusting for diet and physical activity [4]. This association was stronger between mothers with their sons and daughters, and in households with less food security compared to those with greater food security. The prevalence of obesity has been rising in low- and middle-income countries [5]. In Brazil, for instance, the cost of natural foods has increased in recent decades, whereas the prices of processed and ultra-processed foods have decreased, rendering them more accessible and affordable [6]. These types of foods have been associated with food addiction.

Food addiction can be defined as continuous hyper-palatable food consumption, even after meeting energy needs, and despite knowing the negative physical and psychological consequences associated with uncontrolled food intake [7]. A large cohort study showed that the consumption of ultra-processed foods by mothers during the period of child-rearing was associated with a greater risk of developing overweight and obesity in their children during childhood and adolescence [8]. In adults, food addiction has been associated with a worse quality of life, higher body mass index (BMI), the development of diet-related diseases, and the lack of weight loss through traditional treatments. Parental food addiction is associated with food addiction behavior in children, a greater risk of obesity and higher BMI [9].

In addition to unhealthy lifestyle habits, obesity has been associated with psychosocial stress in a bidirectional relationship [10]. Furthermore, increased parental stress has been associated with the development of obesity in their children [11,12]. One possible explanation for the relationship between parental stress and childhood obesity is that highly stressed parents may face greater challenges in purchasing and preparing natural, healthy foods for the family, as well as in encouraging physical activity among their children [11]. Although an association between higher parental stress and childhood obesity has been described, a study involving children up to four years of age found no association between maternal stress during the first year of life and the child’s BMI at ages two to four [13]. Similarly, in parent–child dyads with children aged two to five years, parental stress was not associated with an increased risk of obesity in children [14]. These findings highlight a gap in the literature regarding the impact of maternal stress on children’s BMI z-scores.

There is a positive correlation between the snack intake of parents and their children, indicating that a healthy or unhealthy diet followed by parents is associated with a similar diet followed by their children [15]. This context extends to modifiable factors such as food addiction, as well as maternal BMI, and the stress or food addiction of the children themselves. The home environment, predominantly influenced by the mother, can influence whether a child develops healthier or unhealthier BMI-Z.

However, despite these associations, the mechanisms through which maternal characteristics influence child weight trajectories remain poorly understood, particularly in relation to behavioral and psychological factors like food addiction and stress. Considering the complexity and multifactorial approach of childhood obesity and recognizing that social and environmental factors may impact food addiction, stress, and the development of obesity in the mother–child dyad, we hypothesize that maternal nutritional status of the mother, food addiction, and mental health impact the BMI-Z of the children.

The primary aim of this study was to examine the association between anthropometric parameters, food addiction and stress, in children with obesity and their respective mothers, comparing them to healthy control children and their mothers. Additionally, we aimed to investigate how food addiction, stress, and nutritional status of the mother impact the BMI-Z of the children.

## 2. Materials and Methods

### 2.1. Study Design and Participants

This cross-sectional study was conducted with children aged 6 to 12 years, of both sexes, and their respective biological mothers, recruited from six public schools in João Pessoa city, Brazil. The nutritional status of children was assessed, and they were allocated in two groups according to the anthropometric criteria: (1) Control Group (n = 42), BMI-Z > −1.0 and <1.0 z-score and height for age (HAZ) > −1.0 z-score, and (2) Obesity Group (n = 68), BMI-Z > 2.0 z score and HAZ > −1.0 z-score. Regardless of the nutritional status of the mothers, they were allocated to their children’s respective groups (42 Control, and 68 obesity). Only the biological mothers of the children were included in this study, and children who were primarily cared for by grandparents, aunts, or relatives instead of their mothers were excluded. Those with a history of cardiovascular, renal, neurological, psychiatric or endocrine diseases were excluded from the study. The recruitment and data collection period was from March 2022 to June 2023.

### 2.2. Anthropometric Assessment and Body Composition

Weight was measured in kilograms (kg) using a portable digital scale with a maximum capacity of 150 kg and precision of 100 g (OMRON HEALTHCARE, Osaka, Japan). Height was measured with millimeter precision using a standard portable stadiometer (Alturexata; Minas Gerais, Brazil). All anthropometric measurements were evaluated in standing position according to Lohman [16]. The nutritional status of children was calculated using the AnthroPlus v1.0.2 program (World Health Organization, Geneva, Switzerland). For mothers, the body mass index (BMI) was calculated according to the World Health Organization [17].

Waist (WC) and hip (HC) circumferences were measured in the standing position, with the abdomen relaxed and the arms at the sides of the body, using an inelastic tape to the nearest 1 mm. The waist–hip ratio (WHR) was calculated by dividing the WC by the HC in centimeters. Tricipital, subscapular, suprailiac, and thigh skinfolds were measured using an adipometer (AD1009; Sanny). Absolute and body fat percentage (BFP) were calculated according to Slaughter [18] for children and Jackson and Pollock [19] for mothers. All anthropometric measurements were conducted by a trained evaluator.

### 2.3. Assessment of Socio-Economic Status and Food Addiction

A questionnaire was applied to assess socioeconomic status, gathering information about family income, which was completed by the mothers.

The Yale food addiction scale questionnaire for adults (YFAS) and children (YFAS-C), translated and validated for Brazilian Portuguese [20,21], with Cronbach’s alpha of 0.83 and 0.89, was applied to mothers and children, respectively. To identify the presence or absence of food addiction. Questionnaire scores were calculated based on the total number of symptoms endorsed. The diagnosis required the presence of three or more symptoms, in addition to clinically significant impairment or distress.

### 2.4. Assessment of Stress

The Lipp Stress Symptom Inventory (LSSI), validated for Brazilian Portuguese [22], Cronbach’s alpha = 0. 91, was applied to measure the stress levels in the mothers. Stress scores were calculated by summing the relevant symptom items across time frames (24 h, week, month). Participants were then classified into stress phases (alert, resistance, near exhaustion, exhaustion) based on cut-off criteria. The children completed the Child Stress Scale (CSS) questionnaire, a test developed and validated for use in Brazilian children aged 6 to 14 years. The CSS consists of 35 questions related to reactions generally triggered by stress: physical, psychological, psychological with a depressive component, and psychophysiological. Each question is scored on a Likert scale from 0 (never) to 4 points (always) to record the intensity with which the child experienced the described symptoms and indicate the phases of absence of stress, alertness and resistance, near exhaustion, and exhaustion [23]. Internal consistency of the Brazilian Portuguese CSS was reported as Cronbach’s alpha = 0.85. Both questionnaires were applied and interpreted by a trained psychologist.

### 2.5. Statistical Analysis

A power calculation a priori, with a T-test as the primary outcome, was performed using GPower 3.1.9 (University of Kiel). Considering an effect size of 0.5, a beta of 80%, an alpha of 0.05, and an allocation ratio of 1, the sample size for each group was calculated to be 64, resulting in a total sample size of 128. The power (beta) calculated for a control group with n = 42 and 68 children with obesity achieved a power of 71.4%. Analyses performed a posteriori using linear multiple regression, considering a total sample size of 110, 7 predictors, and an alpha of 0.05, resulting in a power of 83.3%.

The normality of variables was assessed using the Shapiro–Wilk test. Variables with a normal distribution were presented as mean and standard deviation, while variables with non-normal distribution were transformed into log and expressed as mean and confidence interval at 95%. Categorical data were analyzed using the Chi-square test. For the analysis of WC, HC, WHR, BFP, in children, a ANCOVA adjusted for sex and age was used. The correlation between the anthropometric variables of children and mothers were analyzed using Pearson correlation. Linear (hierarchical) and logistic regression analysis were performed to further explore the relationships. The level of statistical significance was set at 5%. All statistical analyses were conducted using SPSS software (IBM version 20.0, Chicago, IL, USA). Participants with missing data were handled by excluding only the affected variables rather than the entire individual, except in regression analysis, in accordance with the SPSS default.

## 3. Results

The study was conducted with 42 children and mothers in the control group and 68 children and mothers in the obesity group. Table 1 summarizes the anthropometric characteristics of the children, their respective mothers, and monthly family income. Regarding the children, no differences were found in the proportion of boys and girls, height, and WHR between the groups. Children with obesity were significantly younger and exhibited higher weight, HAZ, BMI-Z, WC, HC, and BFP compared to the control group. Similarly to children, weight, BMI, WC, HC, WHR, and BFP were significantly higher in the mothers of children with obesity. No differences were found in the age of the mothers, height, and family monthly income between the groups.

Table 2 shows the distribution of food addiction and stress conditions among both children and mothers. Children with obesity showed a higher prevalence of food addiction compared to the control group, with no differences found in stress conditions between the groups. Among the mothers, no difference was found in food addiction prevalence between the groups; however, mothers in the obesity group showed a lower prevalence of stress compared to the control group.

Although mothers of eutrophic children showed a higher prevalence of stress, no significant differences were observed in the distribution of stress phases (alert (33.3% vs. 66.7%), resistance (45.7% vs. 54.3%), near exhaustion (75% vs. 25%), and exhaustion (33.3% vs. 66.7%), respectively) compared to mothers of children with obesity.

Table 3 presents the results of a logistic regression analysis examining the odds of developing food addiction and stress in both children and their mothers. Children with obesity exhibited a 17.9-fold higher chance of developing food addiction compared to the control group. No differences were found in the odds of developing stress in children, and food addiction and stress in their mothers.

Table 4 summarizes the correlations between the anthropometric parameters of children and mothers. The anthropometric parameters of the children were correlated with those of the mothers, including weight, BMI-Z and BMI, WC, HC, and BFP.

Table 5 presents the analysis of multiple regression analysis predicting BMI-Z with predictors for both children and mothers. The first and second models exclusively utilized data from children. Model 2, which includes age, gender, food addiction, and stress condition, explains 18.6% of the variance in BMI-Z. Food addiction is a positive predictor for BMI-Z, while stress condition was not significant in this model. In Model 3, which incorporates data from both the children and the mothers, the significance of food addiction in children decreases. Instead, the BMI of the mother (Beta 0.490) and maternal stress (Beta −0.221) emerge as more important predictors for BMI-Z (adjusted R square 0.468) in children.

## 4. Discussion

The present study showed that children and mothers in the obesity group exhibited higher anthropometric parameters, higher prevalence of food addiction in children, and a lower prevalence of stress in mothers compared to the control group. These findings underscore the importance of maintaining healthy weight and habits in mothers to ensure the adequate nutritional status of their children. The relationship between maternal obesity and childhood can be attributed to the fact that childcare responsibilities typically fall on mothers, leading children to adopt maternal behaviors [24]. In this study, only mothers were included, and they were allocated to groups based on the nutritional status of their children, regardless of their own BMI.

Consistent with previous studies, anthropometric parameters, such as weight, BMI-Z, BMI, WC, HC, and BFP were positively correlated between children and their mothers, highlighting the impact of the dyad in the nutritional status [4,25]. The literature supports that compared with fathers, mothers have a greater impact on children BMI [25], in households with lower food security [4]. In addition, children of fathers or mothers with obesity show increased BMI and WC, but only maternal obesity was associated with increased BFP [26].

Children in the obesity group exhibited a higher prevalence of food addiction, with approximately an 18-fold increased risk of developing food addiction, while this association was not found in mothers. The food addiction and age of children had the most significant impact on BMI-Z among sex and stress. Ultra-processed foods contain highly palatable ingredients, such as high sodium, sugar, and saturated fat, can be addictive, and their consumption has been associated with overweight and obesity in children and adults [8]. Regression analysis was conducted using a hierarchical approach to independently examine the contribution of each block of child and maternal variables to BMI-Z. In Model 1, the child’s non-modifiable variables, such as sex and age, were included. In Model 2, child-level factors related to food addiction and stress were added. The final model (Model 3) incorporated both the child variables and the maternal variables. Model 1 indicated that sex and age significantly influenced BMI-Z. When adjusted for the child’s food addiction and stress (Model 2), food addiction emerged as the most important variable, explaining approximately 19% of the variance in BMI/A. This finding suggests that food addiction is an important characteristic influencing a child’s BMI/A. However, after controlling for maternal BMI, maternal stress, and food addiction (Model 3), food addiction (children) was no longer a significant predictor of child BMI-Z, and the adjusted R^2^ increased substantially from 0.186 (Model 2) to 0.468. This result suggests that maternal characteristics exert a stronger influence on BMI-Z than individual child factors such as food addiction. In Model 3, maternal BMI was the strongest predictor (β = 0.490), while maternal stress showed a negative association with child BMI-Z (β = −0.221), indicating a potential protective effect.

Although the child’s food addiction did not reach statistical significance in Model 3, the data set suggests that it is an important factor in increasing BMI-Z, as higher food addiction was more prevalent among obese children, who were almost 18-fold to exhibit food addiction. However, our findings indicate that, regardless of the child’s food addiction, maternal BMI remains an important predictor of child BMI-Z. This study did not observe a higher prevalence of food addiction among mothers of obese children, however maternal influences, whether behavioral, environmental, or genetic, may have contributed to this outcome. Furthermore, the anthropometric parameters of the child and mother also showed a moderate correlation.

Effective stress management by parents tends to lead to healthier habits when faced with stressors, resulting in healthier eating habits for their children. Parental stress can negatively impact the dietary intake of the children and has been linked to a high risk of childhood obesity [27]. It has been described that maternal stress contributes to more controlled eating practices, a lower intake of homemade foods, and an increase in children consumption of sweets [28]. When mothers experience stress, they may be less attentive to their children’s dietary choices, leading to increased consumption of sweets or unhealthy foods. Additionally, there may be an intimate association between maternal and child stress, whereby the stress of one member of the dyad can influence the stress levels of the other, thus impacting the types of food consumed [29].

However, in the present study, maternal stress was associated with lower child BMI-Z, suggesting a potential protective role. Although a higher prevalence of stress was found among mothers of eutrophic children, the distribution of stress phases did not differ between the groups. Stress in itself is not inherently harmful (eustress); rather, it is chronic stress that can trigger deleterious effects (distress) [30,31]. In this regard, the stress phases did not differ between the groups and were predominantly concentrated in the initial phases (alertness and resistance), which may have contributed to a protective effect for eutrophic children. This finding contrasts with other studies because factors such as financial difficulties serve as stressors for mothers, consequently, influencing the weight gain of the children. A study conducted with pregnant women found that higher household income was associated with lower parental stress and fewer depressive symptoms [32]. Thus, although lower income can be a potential stressor, in our study, income did not differ between the groups, and the population of both groups lived in nearby locations, of the same socioeconomic class. Additionally, ethnicity has been identified as another factor associated with maternal stress and childhood obesity, with higher BMI observed in non-Hispanic black children [33]. Swyden et al. found that mothers with severe or extreme stress reported using more restrictive eating practices compared to those with normal stress levels. Furthermore, regression analysis conducted by the authors indicated that maternal stress and concern about the weight of the children, in addition to the age of children, contributed to increased use of restrictive eating practices [34].

### Limitations and Implications

This study has limitations such as the unmeasured factors that could act as stressors for mothers beyond income. While we measured family income, other factors such as cognitive and physical stress may also contribute to development of perceived stress. The relationship between maternal and child obesity may suggest that genotype is a mediator of the condition, which does indeed exist, but genetic variants that have a large effect on BMI are rare [1].

The sample studied is small, which may limit statistical power and the generalizability of the findings; however, this does not invalidate the associations observed. The sample was composed exclusively of biological mothers and their respective children to enable the examination of potential biological and behavioral pathways linking maternal and child characteristics. Nonetheless, this specific inclusion criterion limits generalizability to broader populations. Additionally, although the instruments used to assess perceived stress and food addiction were previously validated, it is important to note that all psychometric measures have inherent limitations, especially when applied to diverse populations. In this sense, there are different instruments in the literature that assess stress, making comparison between them difficult. Finally, due to the cross-sectional designed of the study, causality cannot be inferred, and the temporal direction of the observed associations remains uncertain. The results of the present study underscore the importance of a multidisciplinary approach that includes family-based interventions for the prevention and treatment of childhood obesity. The positive association between the anthropometric parameters of mothers and their children, along with the finding that maternal BMI is a significant predictor of the child’s BMI-Z score, highlights the need to direct attention and intervention toward mothers (in addition to children)—particularly those who are overweight—as a strategy to reduce childhood obesity.

## 5. Conclusions

In conclusion, the correlation between anthropometric parameters among children and mothers, as well as the BMI of the mother being the most significant predictor for the BMI-Z, indicates that BMI of the mother directly influences the nutritional status of the child more than food addiction itself or the stress status. Although maternal stress is often associated with obesity, our study found an association with lower BMI-Z. Therefore, maternal physical and mental health directly influence the nutritional status of their child, highlighting the importance of considering the mother–child relationship in strategies for preventing and managing childhood and maternal obesity.

## Figures and Tables

**Table 1:** Anthropometric characteristics of children and mothers separated by groups.

	Children				
	Control (n = 42)		Obesity (n = 68)		*p*
Girls n (%)	29	69	34	50	0.050 *
Age (years)	9.78	1.25	9.20	1.18	0.015 **
Weight (kg)	31.24	7.34	49.96	12.04	<0.001 **
Height (cm)	138.27	10.54	141.05	8.99	0.142 **
HAZ (z score)	0.17	0.93	1.22	1.03	<0.001 **
BMI-Z (z score)	−0.32	0.75	2.84	0.75	<0.001 **
WC (cm)	67.61	65.58–69.64	73.54	72.15–74.93	<0.001 †
HC (cm)	80.01	78.72–81.30	85.49	84.61–86.37	<0.001 †
WHR	0.83	0.81–0.86	0.86	0.84–0.88	0.182 †
BF (%)	27.16	24.42–29.91	33.78	31.90–35.65	0.002 †
	Mothers				
Age (years)	36.39	6.45	38.67	7.15	0.108 **
Weight (kg)	67.10	13.42	78.67	15.06	<0.001 **
Height (cm)	159.86	5.80	159.78	6.13	0.948 **
BMI (kg/m^2^)	26.29	5.27	30.76	5.40	<0.001 **
WC (cm)	82.82	11.81	91.40	10.96	<0.001 **
HC (cm)	103.04	11.57	110.54	11.57	0.002 **
WHR	0.80	0.06	0.83	0.06	0.018 **
BF (%)	27.56	6.12	32.37	5.03	<0.001 **
Income					
Income up to 1 salary n (%)	21	56.8	38	63.3	0.519 *
Income above 1 salary n (%)	16	43.2	22	36.7	

* Chi-square. Data are expressed as n (%). ** *T*-test. Data are expressed as mean and standard deviation. † ANCOVA adjusted by, age, sex, and weight. Data are expressed as mean and confidence interval. BMI, Body Mass Index; HAZ, Height-for-age; BMI-Z, Body Mass Index for Age; WC, Waist Circumference, HC, Hip Circumference; WHR, Waist–Hip Ratio; BFP, body fat percentage. Value of the minimum wage in dollars is US$264.37.

**Table 2:** Distribution of children and mothers with food addiction and stress separated by groups.

	Children				
	Control (n = 42)		Obesity (n = 68)		*p*
Food addiction					
Without food addiction	38	97.4	53	77.9	0.006 *
Food addiction	1	2.6	15	22.1	
Stress					
Not stressed	13	41.9	14	29.2	0.243 *
Stressed	18	58.1	34	70.8	
	Mothers				
Food addiction					
Without food addiction	38	97.4	59	93.7	0.647 **
Food addiction	1	2.6	4	6.3	
Stress					
Not stressed	11	31.4	29	53.7	0.039 *
Stressed	24	68.6	25	46.3	

Data are expressed as n and percentage. * Chi-Square Test. Pearson Chi-Square test for food addiction and stress in children are 7.407 and 1.365, respectively. For mother Pearson Chi-Square test for food addiction is 4.258. ** Fisher’s Exact Test.

**Table 3:** Logistic regression analysis of the odds to developing food addiction and stress in children and mothers.

Children	Odds Ratio	95% CI	*p*
Food addiction			
Group with obesity (versus control)	17.910	2.067–155.20	0.009 *
Stress			
Group with obesity (versus control)	1.801	0.666–4.869	0.246 *
Mothers			
Food addiction			
Group with obesity (versus control)	2.854	0.300–27.137	0.361 **
Stress			
Group with obesity (versus control)	0.400	0.157–1.018	0.055 **

* Adjusted by sex and age of children. ** Adjusted by age of mother.

**Table 4:** Correlation between anthropometric parameters of children with their mothers.

	Children					
Mothers	Weight (kg)	HAZ (z Score)	BMI-Z (z Score)	WC (cm)	HC (cm)	BFP (%)
Weight (kg)	0.433 †	0.370 †	0.487 †	0.421 †	0.437 †	0.420 †
BMI (kg/m^2^)	0.444 †	0.259 †	0.473 †	0.429 †	0.448 †	0.432 †
WC (cm)	0.451 †	0.314 †	0.454 †	0.461 †	0.437 †	0.454 †
HC (cm)	0.392 †	0.302 †	0.442 †	0.410 †	0.404 †	0.401 †
BFP (%)	0.311 †	0.223 *	0.398 †	0.285 †	0.330 †	0.401 †

Pearson correlation. * *p* < 0.05 and † *p* < 0.01. BMI, Body Mass Index; HAZ, Height-for-age; BMI-Z, Body Mass Index for Age; WC, Waist Circumference; HC, Hip Circumference; WHR, Waist–Hip Ratio; BFP, body fat percentage.

**Table 5:** Multiple regression analysis predicting body mass index for age with predictors of children and mothers.

Variables	B	SE for B	Beta Coefficient	*p*	*p* Value (ANOVA)	Adjusted R Square
Model 1 (children)						
Constant	4.795	1.596		0.004	0.008	0.112
Sex	0.900	0.403	0.259	0.029		
Age	−0.378	0.168	−0.261	0.028		
Model 2 (children)						
Constant	4.998	1.575		0.002	0.002	0.186
Sex	0.764	0.389	0.220	0.054		
Age	−0.427	0.163	−0.295	0.011		
Food addiction	1.655	0.631	0.299	0.011		
Stress condition	0.212	0.401	0.060	0.599		
Model 3 (children and mother)						
Constant	0.912	1.552		0.559	<0.001	0.468
Sex (children)	0.378	0.324	0.109	0.249		
Age (children)	−0.354	0.133	−0.244	0.010		
Food addiction (children)	1.000	0.536	0.181	0.067		
Stress condition (children)	0.073	0.326	0.020	0.824		
BMI (mother)	0.140	0.028	0.490	<0.001		
Food addiction (mother)	0.635	0.687	0.089	0.359		
Stress condition (mother)	−0.751	0.321	−0.221	0.023		

Sex: (0 girls, 1 boys). Food addiction: (0 without food addiction, 1 food addiction). Presence of stress: (0 without stress, 1 stressed). Note: B = unstandardized coefficient; SE = standard error; Beta = standardized coefficient; *p* = *p*-value; Adjusted R^2^ = adjusted coefficient of determination.

## Data Availability

The raw data supporting the conclusions of this article will be made available by the authors on request.

## Abbreviations

The following abbreviations are used in this manuscript:

BMI	Body Mass Index
BMI-Z	Body Mass Index for Age
HAZ	Height For Age
WC	Waist Circumference Waist
HC	Hip Circumference
WHR	Waist–Hip Ratio
BFP	Body Fat Percentage
YFAS	Yale Food Addiction Scale Questionnaire For adults
YFAS-C	Yale Food Addiction Scale Questionnaire For Children
LSSI	Lipp Stress Symptom Inventory
CSS	Child Stress Scale
